# Supplementary material for: All-optical steering on the proton emission in laser-induced nanoplasmas
Source: Nat Commun. 2024 Jun 17;15:5150. doi: 10.1038/s41467-024-49569-3 (PMC11183200; doi:10.1038/s41467-024-49569-3)
Supplement: Supplementary file 1 — Supplementary Information [file 41467_2024_49569_MOESM1_ESM.pdf]

Supplementary Information to
"All-optical steering on the proton emission in laser-induced nanoplasmas"

Fenghao Sun[1,2], Qiwen Qu[1], Hui Li[1,*], Shicheng Jiang[1,*], Qingcao Liu[3], Shuai Ben[4], Yu Pei[4], Jiaying Liang[4], Jiawei Wang[1], Shanshan Song[1], Jian Gao[1], Weifeng Yang[4,5], Hongxing Xu[1], Jian Wu [1,6,7 *]

[1]*State Key Laboratory of Precision Spectroscopy, East China Normal University, Shanghai 200241, China*

[2]*School of Information Science and Engineering, Harbin Institute of Technology, Weihai 264209, China*

[3]*College of Science, Harbin Institute of Technology, Weihai 264209, China*

[4]*School of Physics and Optoelectronic Engineering, Hainan University, Haikou 570228, China*

[5]*Center for Theoretical Physics, Hainan University, Haikou 570228, China*

[6]*Chongqing Key Laboratory of Precision Optics, Chongqing Institute of East China Normal University, Chongqing 401121, China*

[7]*Collaborative Innovation Center of Extreme Optics, Shanxi University, Taiyuan, Shanxi 030006, China*

[*]*hli@lps.ecnu.edu.cn*
[*]*scjiang@lps.ecnu.edu.cn*
[*]*jwu@phy.ecnu.edu.cn*

I. Classical model for calculating the far-field momentum distributions

In the present work, the ejected protons from the gold nanospheres were detected using a single-shot VMI apparatus. The far-field momentum distributions and the initial state of the nanoscale spatial distributions for the protons can be linked by a simple classical model involving the ionization and the classical motion of the charged particles. This method has been implemented in our previous work to explore the ion emission property in dielectric nanosystems[1,2]. In the first step, FDTD calculations were carried out to estimate the induced near-field distributions around the nanosystem (Supplementary Fig. 1 (a)). The initial spatial distribution of the produced protons was estimated according to this near field profile, considering a reasonable ionization rate as a function of the local field strength. In the second step, the generated ions were flying towards the detector where their behaviors are governed by the Coulomb interactions. For simplicity, the calculations were carried out by considering the repulsive interactions between the localized nanoplasma and the H^+ ions in a two-dimensional plane, as shown in Supplementary Fig. 1(b). The gold nanosphere was placed at the origin, i.e. at (0, 0). The ion density function gradually decreases as they flying away from the origin. At the 800 nm excitation condition, two effective positive charges (representing the equivalent charge state of the localized plasma) were placed at the positions of (-45 nm,-2 nm) and (45 nm,-2 nm) based on the calculated enhanced near field distribution. After setting the position of the effective positive charges, the following dynamics of the H^+ ions were calculated based on the Newtonian equations of motion. The dynamics of all the ions were calculated at time steps of 200 fs and the momentum at t = 100 ps was presented when the ion distribution reached a steady state (Supplementary Fig. 1 (c)). The simulation at 400 nm excitation is operated in a similar way. One only needs to change the initial ion distributions and the corresponding effective charge positions for the localized nanoplasma based on the near field distributions.

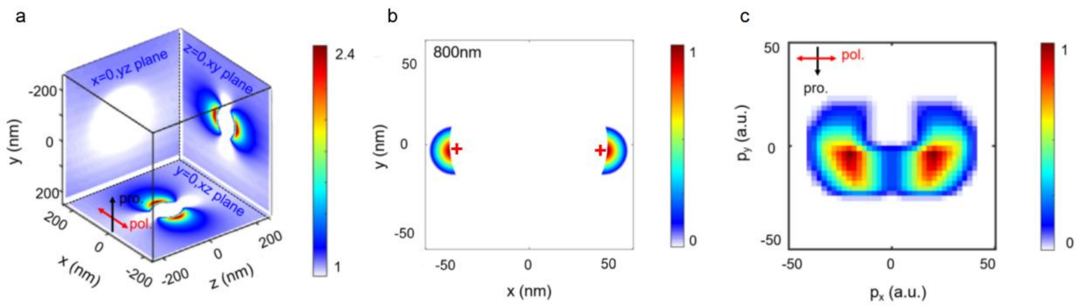

Supplementary Figure 1. FDTD simulation and classical proton momentum calculation under 800nm laser excitation. (a) The calculation results of the near field |E| distributions based on FDTD for 100 nm gold nanospheres interacting with femtosecond laser pulses 800 nm. (b) The initial spatial distributions of the H^+ ions from gold nanospheres excited by femtosecond laser pulses at 800 nm. The red plus signs represent the effective charges for the localized plasmas. (c) Calculated far-field momentum distributions of protons ejected from gold nanoparticles irradiated by 800 nm laser.

II. Data sorting based on the single-shot ion yield

In the single-shot experiment, the interaction results between individual nanoparticle and isolated femtosecond pulse can be obtained explicitly. Due to the fact that the focusing laser beam exhibits a Gaussian profile, and that the size of the nanoparticles is orders of magnitude smaller than the focusing spot, each nanosphere could experience a distinct effective intensity depending on the specific interaction position at the laser focus. In this case, the total ion yield obtained for each shot could differ largely. However, this offers a handy way to sort the data since the total ion yield can be related to the effective intensity for each measurement[1,2]. Our data were classified by the total ion yield, equivalent to being sorted by laser intensity. This classification method is similar to the scheme based on single-shot electron counts in the previous work[3].

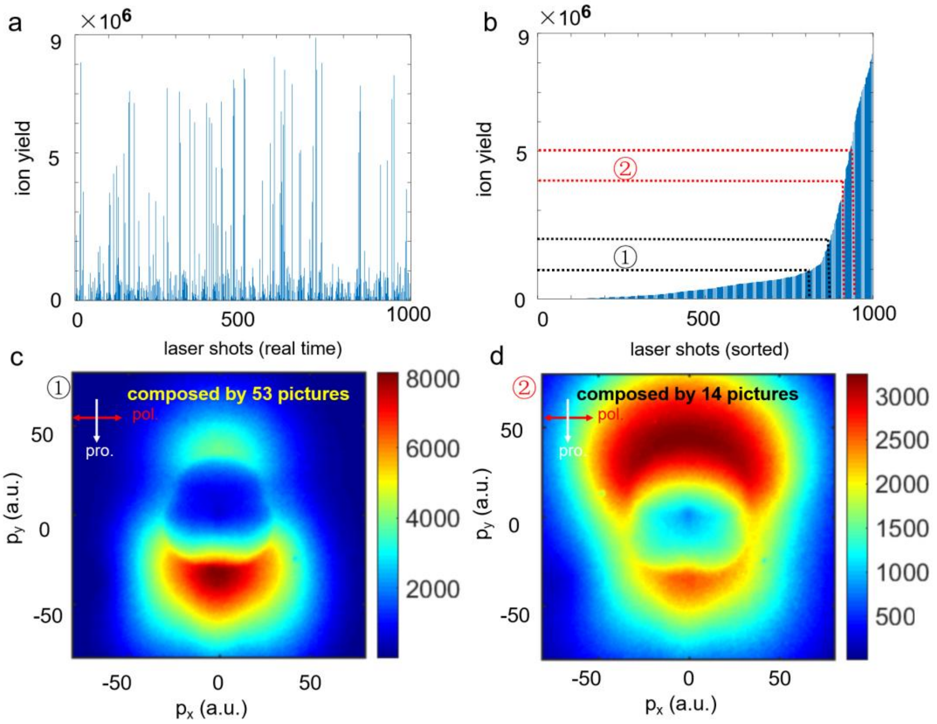

Supplementary Figure 2. Schematic of a histgram classification method. (a) The ion yields of 1000 laser shots in real time. (b) Sorted histgram of ion yields of the 1000 laser shots. (c,d) are the H^+ ion momentum distributions selected by ion yields and corresponding to the interval (①,②) indicated in Supplementary Fig. 2(b), respectively.

Supplementary Fig. 2 (a) shows the ion yields for 1000 laser shots, exhibiting a random distribution. However, if we sort these single-shot data by the total ion yield, a clear histogram can be obtained as being presented in Supplementary Fig. 2 (b). Different regions in the histogram were selected, where the single-shot images within the region were integrated. By doing this we can get a momentum distribution with better statistics for a specified intensity region. Supplementary Figs. 2 (c,d) show the integrated momentum distributions of H^+ for the two distinct regions indicated in Supplementary Fig. 2 (b). The resulting distributions are different for two effective laser intensities. The result obtained for Region ① shows a foward focusing effect with more ions being detected in the -y direction, while the image for Region ② exhibits an opposite tendency, such that more ions are ejected towards the backward

direction. These differences are governed by the excitation laser intensities.

Supplementary Fig. 3 shows a set of sorting result of the proton momentum distributions at small ion counts intervals, corresponding to a set of laser-intensity-dependent result.

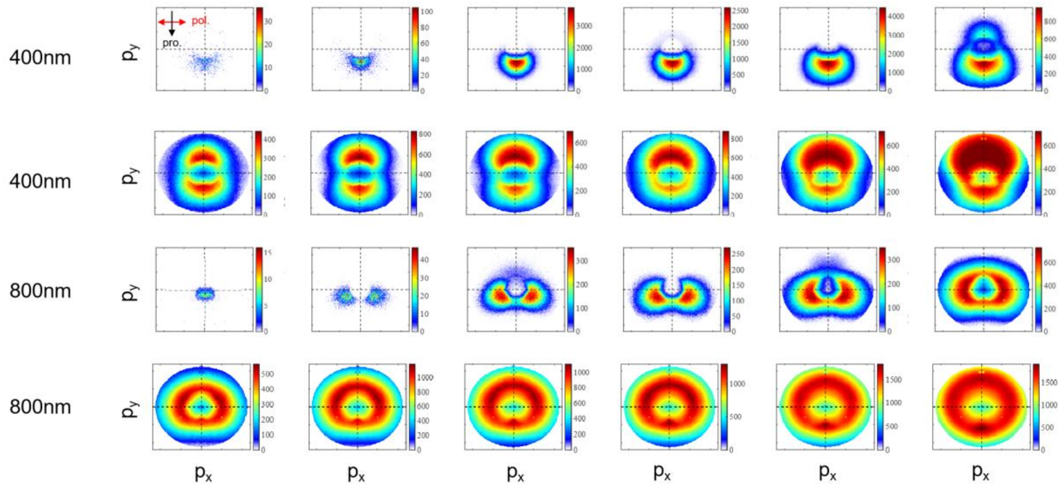

Supplementary Figure 3. The momentum distribution diagram at small ion counts interval for protons. The top two rows correspond to experimental data under 400nm laser excitation, while the following two rows represent experimental data of 800nm.

III. 3D tomographic imaging

In order to directly visualize the three-dimensional (3D) momentum distributions for protons, the projected momentum images under the excitation of 18 different laser polarization directions (at 10 degree interval) were detected. The momentum distributions obtained for the 18 angles were placed in a 3D matrix, as a function of p_x, p_y and the polarization angles (shown in Supplementary Fig. 4).

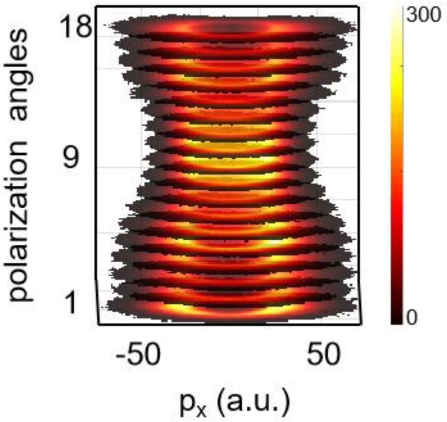

Supplementary Figure 4. The 3D matrix obtained at 18 polarization angles projecting onto the coordinations of p_y and polarization angle.

The reconstruction of the 3D distribution from the 2D data were operated using the iradon function[4] library provided by Matlab. We take the reconstruction of a prototype image as an example, as shown in Supplementary Fig. 5. After projecting a 2D image obtained at different positions/angles, the 1D projection lines at different projection angle can be obtained as shown in Supplementary Figs. 5 (b) and (d). Supplementary Figs. 5 (c) and (e) are the images restored by iradon function with projection angle intervals of 1 degree and 20 degrees, respectively. It can be seen that better imaging quality can be achieved by using a smaller angle interval during the reconstruction algorithm.

Similarly, a 3D distribution can be reconstructed from several 2D images obtained at different angles. In our experiment, we obtained a 3D matrix of 244×244 ×18 from measurements at 18 polarization angles.The matrix were divided into 244 groups such that each 244×18 unit contains the 1D distributions for 18 angles. Then we ran the Radon transformation based on projecting the image at 10-degree interval where a 2D distribution can be obtained. Using the iradon function, a single 244×18 matrix can be transformed to a 244×244 matrix, corresponding to a slice within the 3D distribution, as shown in Supplementary Fig. 6 (a). This step was repeated and finally a 3D distribution can be obtained by stacking the 244 slices together, as shown in Supplementary Fig. 6 (b).

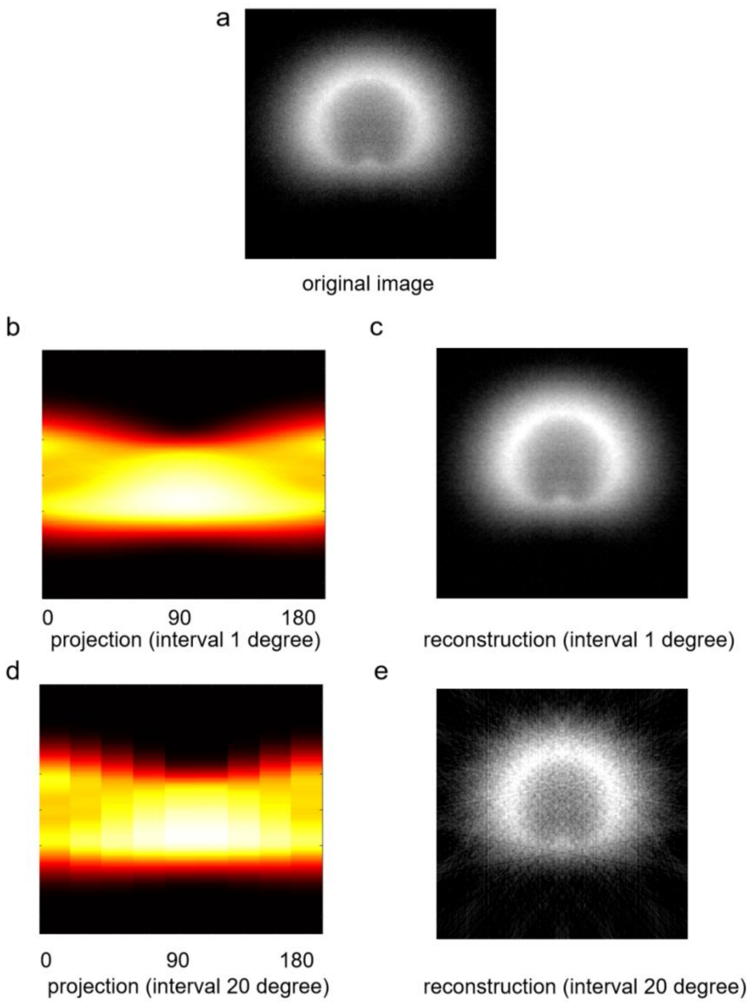

Supplementary Figure 5. Data reconstruction under different projection intervals. (a) The original data, (b, d) are the projected images with different projection intervals, (c, e) are the reconstructed images for (b) and (d), respectively.

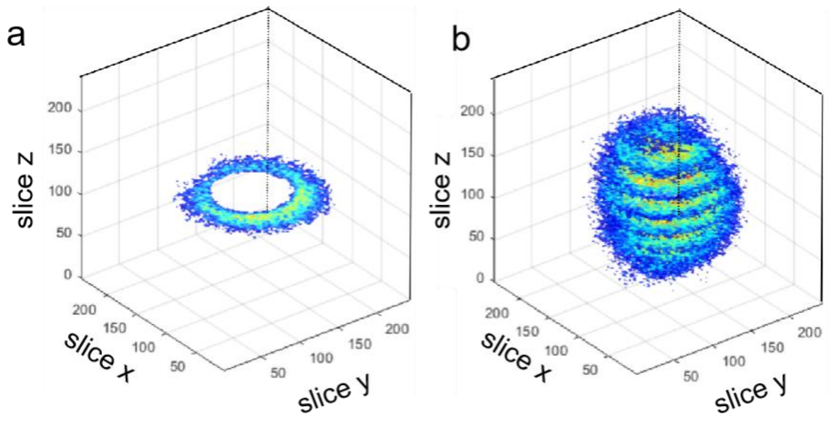

Supplementary Figure 6. 3D imaging obtained by slice plot. (a) Single slice

reconstructed by a 244×18 matrix, (b) The 3D distribution obtained by stacking several slices.

IV. Time of flight spectra and momentum distributions obtained for other ion species

Supplementary Fig. 7 shows the single-shot time of flight (TOF) spectra obtained for the 100 nm gold nanospheres excited by intense femtosecond pulses at 400 nm. The results obtained at 800 nm are similar. Besides the H^+ signal at m/q=1, a wide distribution can be recognized around m/q=12, which can be contributed to the CH_n^+ groups (n=0,1,2...). The yield of the broad peak near m/q=12 is about 5% compared to the yield obtained for proton. The momentum distributions are obtained for the broad peak around m/q=12 and are explicitly shown in Supplementary Figs. 8 c and d. The property is to some extent similar to what we obtained for H^+ (as shown in Supplementary Figs. 8 a and b).

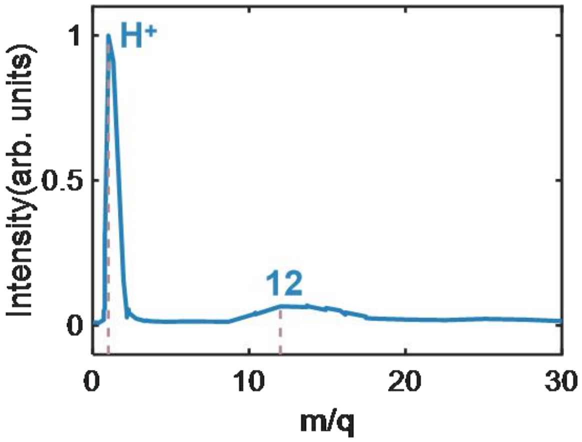

Supplementary Figure 7. Single-shot TOF spectra obtained for the 100 nm gold nanospheres excited by intense femtosecond pulses.

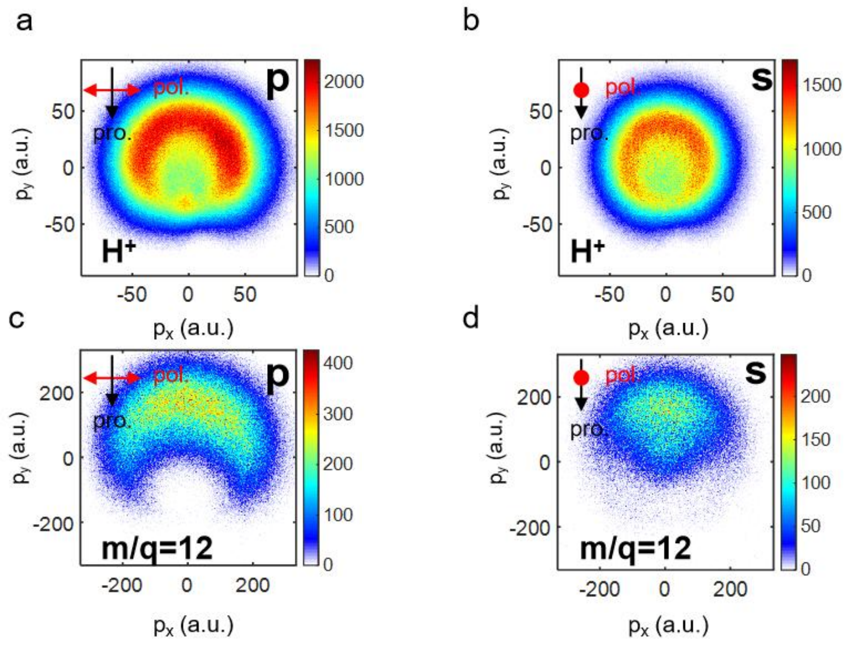

Supplementary Figure 8. Integrated momentum distribution of different nuclear mass ratios and laser polarizations. Integtrated momentum distributions obtained for protons (a,b) and for the broad peak at around m/q = 12 (c,d). p/s represents that the laser polarization direction is parallel/perpendicular to the detector plane.

V. Near-field real-space and far-field momentum distributions obtained by modified PIC method

The modified PIC simulation of the experimental results was calculated through two programs. The ultrafast ionization kinetics during the femtosecond laser duration were calculated for a period of 100 fs, with a time accuracy of 2 as. The evolution time to reach a steady state in the momentum space is about 100 ps. The field-free momentum distribution simulation is obtained through classical Coulomb evolution with a time accuracy of 200 fs. The real-space charge density generated by the first ionization step can be transformed into a two-dimensional matrix and be used as the input file for the second step Coulomb evolution. Supplementary Figs. 9 (a-e) are the near-field real-space proton distributions calculated by PIC under excitations at 400 nm for different laser intensities. Supplementary Figs. 9 (f-j) are the calculated far-field momentum distributions obtained from Coulomb classical simulations which

was introduced in Section I. Supplementary Figs. 9 (k-o) are the corresponding experimental data.

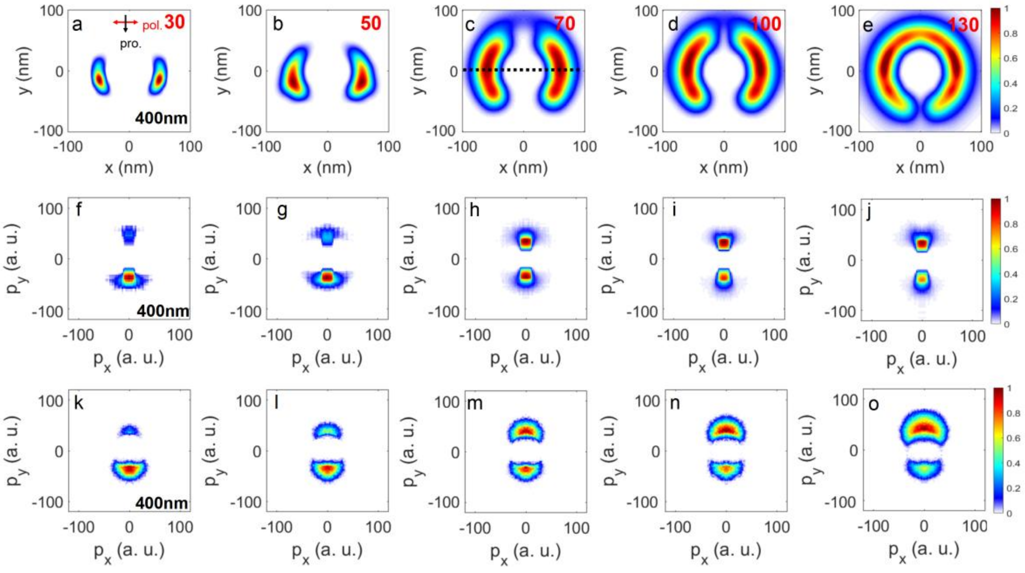

Supplementary Figure 9. Experimental and theoretical intensity-dependent proton momentum distribution. The intensity-dependent near-field real-space proton distributions at (a-e) 400 nm. The unit for the value indicated in the upper right corner of each image is TW cm^{-2}. (f-j) and (k-o) are the calculated far-field momentum distributions and corresponding measured single shot momentum distributions, respectively.

A different intensity-dependent feature in the far-field momentum distributions can be recognized. As shown in Supplementary Fig. 9, the calculated near-field and far-field distributions at 400 nm switched from the forward to the backward enhancement within a very small intensity range, such as shown in Supplementary Figs. 9 (b, c) and the corresponding far-field distributions in Supplementary Figs. 9 (g, h). The red values indicated in the figures are the laser intensities in unit of TW cm^{-2}. The forward to backward swithes within a range of 20 TW cm^{-2}. However, the case is different for the 800 nm excitations. As is shown in Fig. 5 in main manuscript, a slightly forward to backward change can be recognized when the intensity increases from ~30 to 130 TW cm^{-2}. The switching occurs in a much wider intensity range. This also contributes to a direct forward and backward enhancement switch observed for the 400 nm excitations, and a gradually change from forward to sideward, and to

backward for the 800 nm excitations, as the laser intensity is increased.

References:

1. Sun, F. et al. Single-shot imaging of surface molecular ionization in nanosystems. *Nanophotonics* 10, 2651 (2021).
2. Wang, J. et al. Surface molecular ionization imaging of gold nanocubes. *Opt. Express* 31, 9678 (2023).
3. Powell, J. A. et al. Interplay of pulse duration, peak intensity, and particle size in laser-driven electron emission from silica nanospheres. *Opt. Express* 27, 27124 (2019).
4. Radermacher M. Radon transform techniques for alignment and three dimensional reconstruction from random projections. 1997.
